# Emotional belonging and cultural identity: a systematic review of collective emotions among indigenous and minority groups

**DOI:** 10.3389/fsoc.2026.1766705

**Published:** 2026-03-17

**Authors:** Margit Julia Guerra-Ayala, Emma Lourdes Durand-Gómez, Edith Cari Checa, Rildo Paul Tapia Condori, Bresia Maryory Abarca Cari

**Affiliations:** 1 Universidad Tecnológica del Perú, Arequipa, Peru; 2 Escuela de Educación Superior Pedagógica Pública Arequipa, Arequipa, Peru; 3 Universidad Andina Néstor Cáceres Velásquez, Juliaca, Peru; 4 Universidad Católica de Santa María, Arequipa, Peru

**Keywords:** affective sociology, belonging, collective emotions, cultural identity, interculturality, moral recognition, resilience, social cohesion

## Abstract

**Introduction:**

Collective emotions are central to understanding how belonging, identity, and social cohesion are constructed within indigenous, migrant, and minority communities. Beyond individual feelings, they function as cultural and moral frameworks that sustain recognition and collective action. This systematic review addresses the following research question: How do collective emotions contribute to processes of cohesion, resistance, and cultural reconstruction among Indigenous and minority groups across institutional contexts?

**Methods:**

Following PRISMA 2020 guidelines, this systematic review analyzed empirical and theoretical studies published between 2015 and 2025 in Scopus, Web of Science, and ScienceDirect.

**Results:**

Seventy-four studies were included, examining emotions linked to identity, resistance, and social reconstruction across intercultural contexts. The synthesis identified eight dominant emotional mechanisms: nostalgia, pride, shame, guilt, hope, compassion, resilience, and resentment, emerging across educational, migratory, and political settings. These emotions shape cohesion, moral repair, and symbolic resistance, revealing how collective emotions sustain community life and redefine social justice.

**Discussion:**

Findings indicate that collective emotions function as social practices organizing belonging, agency, and recognition. They mediate between memory and power, showing that emotional climates underpin cultural resilience and political transformation.

## 1 Introduction

Collective emotions constitute one of the most dynamic and complex fields of contemporary sociology. These contributions highlight the central role of collective emotions in structuring communal responses to mobility, inequality, and social transformation, particularly through processes of cohesion, resistance, and cultural reconstruction. Far from being mere individual reactions, emotions have become social structures of feeling, in the sense proposed by Williams (1997) articulating the links between memory, identity, and power. Among Indigenous, migrant, or minority communities, these emotions act as an invisible fabric that sustains belonging and symbolic continuity, defining the moral boundaries of the “we” in the face of globalization, colonization, or structural exclusion.

Over the past decade, studies on collective emotions have shown increasing interdisciplinary convergence, calling for a systematic synthesis from sociology. In this regard, Carson et al. (2024) demonstrate that cultural identity and emotional belonging are determinants of collective wellbeing, while warning that the literature continues to privilege psychological approaches over sociological and intercultural perspectives. This imbalance highlights the need for conceptual frameworks that recognize the cultural and colonial specificities of emotional experience. Likewise, Schulte and Trinn (2024) proposed the Forest-Fire Model of Cultural Identity Conflict Escalation, showing how emotions such as pride, anger, or resentment act as self-organizing forces capable of triggering and amplifying identity conflicts. Their model reveals that collective emotions not only result from conflict but also drive and structure it, providing empirical support for the idea that they constitute cultural and moral organizing nuclei sustaining social action.

From a conceptual perspective, the role of collective emotions in shaping identity and belonging has become central in recent sociocultural studies. Emotions, rather than biographical responses, are technologies of connection that mediate between the individual and the community (Illouz, 2019). Their circulation—through symbols, discourse, and everyday practices—produces solidarities, hierarchies, and shared memories. Ahmed (2014) emphasizes that emotions are performative: they do something in the social world, as their repeated expression produces material effects of inclusion or exclusion. Similarly, Barbalet (2002) conceives of emotions as foundations of moral action and social order, while (Illouz, 2019) demonstrates that affectivity has become a structuring principle of modern institutions. These perspectives converge on a key idea: emotions are not premodern residues but contemporary mechanisms of social agency that define how individuals perceive and legitimize their belonging.

The theoretical framework underpinning this analysis integrates contributions from the sociology of emotions with critical and cultural perspectives. From Williams (1997) notion of “structures of feeling” to Ahmed (2014) theory of social affectivity, and Illouz (2019) conceptualization of emotional capitalism, the study of emotions has shifted from the individual to the relational level. Collective emotions are understood as mediations linking personal experience with cultural narratives and political structures. Barbalet (2002) highlights that emotions not only motivate social action but also regulate the hierarchies and moral values that sustain it. While affect theory conceptualizes affect as a pre-discursive and pre-personal intensity that precedes social articulation (Massumi, 2021, 1995) and distinguishes it from emotion as its culturally mediated and narrativized expression (Anderson, 2013), this review adopts the perspective of the sociology of emotions. Accordingly, it focuses on collective emotions as socially structured, publicly articulated, and institutionally embedded phenomena that organize belonging, recognition, and moral boundaries within communities. For conceptual consistency, the term “collective emotions” is employed throughout the manuscript.

In both Latin American and global contexts, recent research has shown that collective emotions play a key role in cultural reconstruction, linguistic revitalization, and processes of symbolic justice. Authors such as Nikielska-Sekuła (2023), Heid et al. (2022), Boric Bargetto et al. (2021), and Sanabria et al. (2024) emphasize that hope, nostalgia, and empathy strengthen interethnic bonds and act as emotional resources for collective resilience.

Similarly, Branch (2021) and Jardim and Marques da Silva (2021) demonstrate that ethnic and identity pride fosters academic agency and integration in multicultural educational contexts. However, structural limitations persist, as most studies focus on Euro-American regions, with a predominance of qualitative methodologies and limited triangulation or application of sociological frameworks. This has led to a fragmented field and a lack of systematic evidence connecting collective emotions with processes of cultural belonging and moral reconstruction.

Recent scholarship has further deepened the understanding of diaspora and interethnic community formation. Demir's work on Kurdish diasporic identity highlights how diasporic communities construct transnational belonging and political identity through narrative practices and ongoing engagement with struggles in the homeland (Demir, 2015, 2012). Similarly, ethnographic work on Mapuche spiritual and communal practices illustrates how emotional solidarities and cultural rituals shape communal identity and resistance in colonial and postcolonial contexts (Bacigalupo, 2016). These perspectives reinforce the argument that collective emotions are not merely expressive phenomena but structuring forces in the reconstruction of communal life.

Despite the growing body of interdisciplinary research on collective emotions, the literature remains conceptually fragmented and unevenly distributed across psychological, cultural, and political analyses (Carson et al., 2024; Schulte and Trinn, 2024). There is, to date, no systematic synthesis that examines how collective emotions operate specifically within Indigenous and minority communities across institutional contexts, nor how these emotions mediate processes of recognition, belonging, and resistance. In a global context marked by intensified mobility, resurgent nationalism, and enduring colonial legacies (Sánchez-Sibony, 2024; Castelo Branco and Gouvêa, 2025), such a synthesis is theoretically and politically urgent. This review responds to that gap by offering a structured sociological analysis of collective emotions in intercultural settings.

Within this framework, the present systematic review aims to analyze the empirical and theoretical evidence regarding the role of collective emotions in the construction of cultural identity and belonging among Indigenous and minority groups. Specifically, it seeks to identify the predominant emotions shaping the affective life of communities, examine the social and institutional contexts in which these emotions manifest, and understand the sociocultural mechanisms through which they operate, revealing their structuring function in group identity and the dynamics of belonging and cultural recognition.

## 2 Methodology

This systematic review was conducted in accordance with the PRISMA 2020 guidelines, with the aim of ensuring a rigorous analysis of empirical and theoretical evidence related to the emotional and cultural dimensions of belonging in migratory contexts. The methodological process comprised three main stages: (a) the search and retrieval of studies from three major indexed databases relevant to the social sciences—Web of Science, Scopus, and ScienceDirect; (b) screening and eligibility assessment using PICOS criteria (Population, Interest, Context, Outcome, and Study design), summarized in Table 1; and (c) a narrative and interpretative synthesis of the findings.

**Table 1:** PICOS criteria.

**Element**	**Definition (adapted to the sociological domain)**	**Application in the study**
P – Population	Indigenous, migrant, or minority groups (ethnic, linguistic, cultural).	Indigenous peoples, linguistic minorities, migrant populations in processes of acculturation, or minority groups experiencing exclusion, uprooting, or subordination.
I – Interest *(instead of Intervention)*	Collective emotions related to belonging or cultural identity.	Pride, nostalgia, shame, hope, resentment, etc.
C – Context/Comparison	Social or institutional contexts where emotions emerge (education, policy, migration, language, activism).	Comparison between different contexts or levels (micro, meso, macro).
O – Outcomes	Forms of integration, resistance, or identity reconstruction mediated by collective emotions.	Sociological mechanisms of cohesion, exclusion, or cultural resistance.
S – Study design	Empirical or theoretical studies with a sociological focus, published between 2010 and 2025.	Qualitative, quantitative, or mixed peer-reviewed studies.

The search equations were adapted to the specific syntax of each database, combining Boolean operators and thematic descriptors related to emotions, cultural identity, and migration. The search strategy (see Table 2) was based on controlled descriptors from the UNESCO Thesaurus of Sociology and MeSH terms associated with collective emotion, cultural identity, belonging, and ethnic minority, adjusted to the syntax of each database.

**Table 2:** Search equations.

**Database**	**Search terms**
Web of science	(“collective emotion” OR “emotional belonging” OR “cultural pride” OR “cultural nostalgia”) AND (indigenous OR migrant OR “ethnic minority” OR “language minority” OR “first nations”) AND (“cultural identity” OR “social identity” OR belonging OR acculturation) AND (sociology OR “social theory” OR community OR education)
Scopus	(collective emotion OR emotional belonging OR cultural pride OR cultural nostalgia) AND TITLE-ABS-KEY (indigenous OR migrant OR “ethnic minority” OR “language minority”) AND TITLE-ABS-KEY (“cultural identity” OR belonging OR acculturation) AND PUBYEAR > 2010 AND PUBYEAR < 2026
ScienceDirect	(“collective emotion” OR “emotional belonging” OR “cultural pride”) AND (indigenous OR migrant OR “ethnic minority”) AND (“cultural identity” OR belonging) AND sociology

Adapted search strategies were adjusted to the syntax of each database, following UNESCO Sociology Thesaurus and MeSH terminology.

The systematic search yielded 105 records from Web of Science, 36 from Scopus, and 79 from ScienceDirect, resulting in a total of 220 references. After automated and manual duplicate verification, one duplicate was removed, leaving 219 unique records for screening. Of these, 145 were included, 62 were excluded, and 12 were initially categorized as “Maybe” due to thematic or methodological ambiguity; these were reassessed during the full-text screening phase. Ultimately, 145 studies were selected for full-text review, representing 66.2% of the total evaluated.

A data extraction table was designed to systematically record key variables from each study, including authorship, year, context, population, type of emotion analyzed, and main findings.

Empirical studies—qualitative, quantitative, or mixed methods—as well as theoretical papers addressing collective emotions in relation to cultural identity and/or the sense of belonging among ethnic, linguistic, migrant, or Indigenous minorities were included. Studies conducted within social, institutional, or cultural contexts (micro, meso, or macro levels) and grounded in a sociological framework of emotions were considered eligible. Texts were required to be written in English or Spanish, published between 2010 and 2025, and appear in peer-reviewed journals. Exceptionally, empirical book chapters or monographs published by recognized academic presses (e.g., Elsevier, Routledge, Springer, Taylor & Francis) presenting qualitative, narrative, ethnographic, or quantitative analyses of cultural identity, belonging, or integration in multicultural or migratory contexts were also included.

Excluded were strictly clinical or psychological studies focused on individual-level interventions lacking a sociological framework of emotions, as well as prior systematic reviews addressing only wellbeing or mental health without examining collective emotions or cultural identity. Gray literature not indexed in academic databases (theses, reports, non-peer-reviewed proceedings) was also excluded, together with studies centered exclusively on individualized emotions (e.g., personal depression or anxiety) without collective or social anchoring, and papers lacking methodological descriptions or defined populations, such as philosophical essays or political analyses without empirical evidence. The inclusion–exclusion process was reviewed independently by two researchers, and discrepancies were resolved through consensus, ensuring inter-rater reliability.

During the full-text review phase, some studies were excluded due to lack of access to the full text, despite exhaustive searches across institutional repositories, academic databases, and open-access platforms. These cases were coded in Rayyan under the category “Full text not accessible (paywalled).” Following PRISMA 2020 guidelines, author contact was considered optional and applied only in cases of high thematic relevance or uniqueness. When access remained unavailable, the records were excluded and transparently reported in the PRISMA flow diagram under “Full-text articles excluded, with reasons.”

The management, screening, and coding process was conducted using Rayyan Premium (Rayyan Systems Inc., Cambridge, MA, United States), a specialized tool for systematic reviews that ensures transparency and reproducibility while minimizing inclusion bias. Risk of bias assessment was performed through cross-review between evaluators, considering methodological transparency, theoretical validity, and the adequacy of the sociological framework. No studies were excluded due to publication bias, given the exploratory nature of the review. Narrative synthesis techniques were applied, integrating findings through thematic analysis and cross-comparison of emotional patterns, contexts, and sociocultural mechanisms.

**Figure 1:**
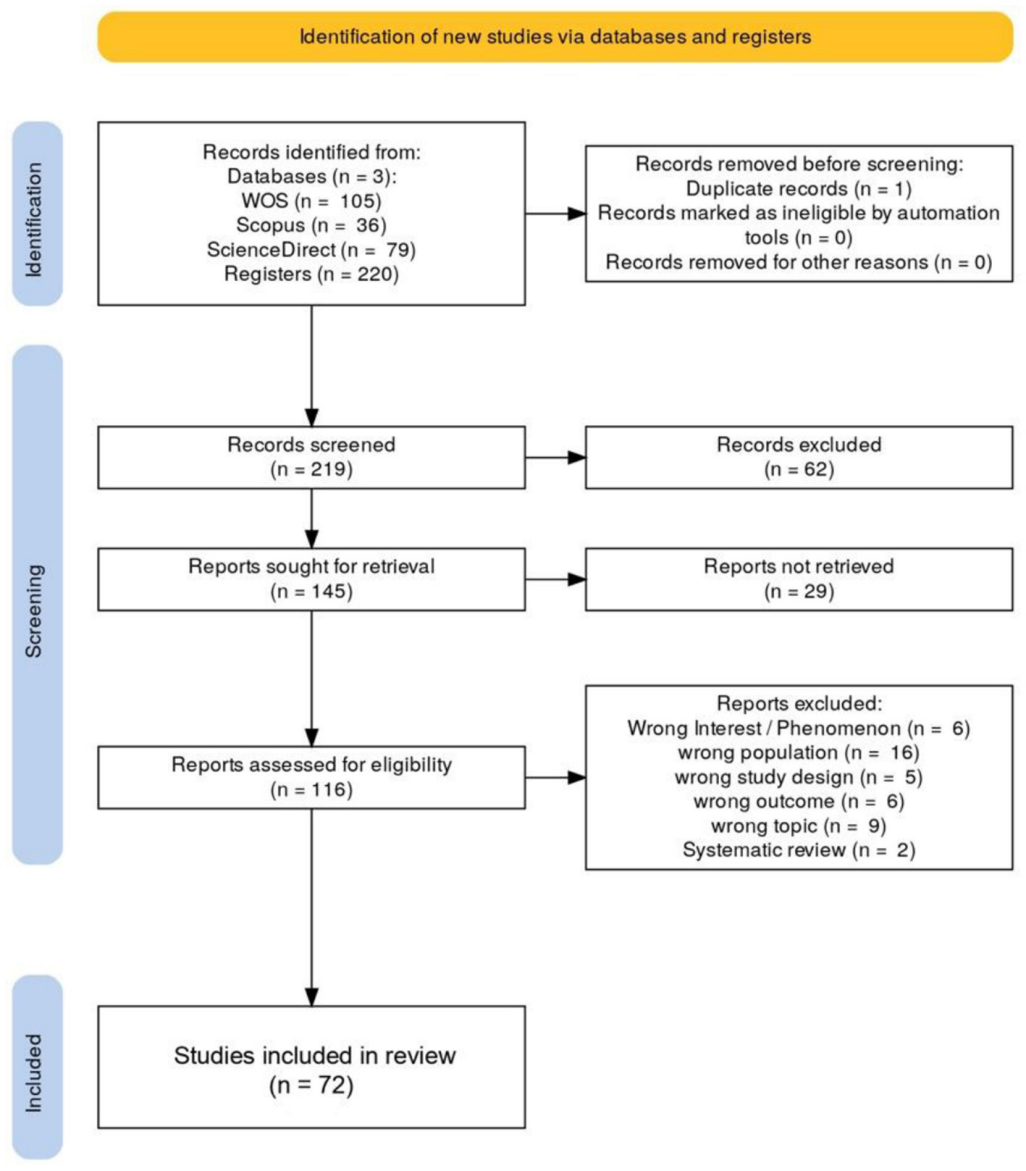
PRISMA flow diagram.

All phases of screening, tagging, and decision logging were documented on the platform and exported for final analysis following PRISMA guidelines (Figure 1). The study was not registered in PROSPERO, given its sociological orientation and the platform's biomedical focus. Nonetheless, the methodological protocol was documented and rigorously followed in accordance with PRISMA 2020 standards.

All records, inclusion/exclusion decisions, and files exported from Rayyan are available upon reasonable request to the research team, ensuring full transparency and reproducibility of the process.

## 3 Results

Of the total 74 articles included, 22 explicitly addressed the most studied collective emotions, 32 focused on the social and institutional contexts in which these emotions emerge, and 38 explored the sociocultural mechanisms through which they shape group identity. This classification, summarized in Table 3, ensures a comprehensive and comparative sociological coverage of the phenomenon.

**Table 3:** Categorization of the included studies according to analytical objective.

**Category**	**Objective**	**No. of articles**	**Description**
1. Most studied collective emotions	Identification of predominant emotions (e.g., pride, hope, shame, nostalgia, resentment).	22	Studies that explicitly describe or analyze collective emotions.
2. Social and institutional contexts	Identification of the settings where emotions are studied (education, migration, language, activism, public policy).	32	Studies that situate the phenomenon within institutional or community environments.
3. Social function	Identification of sociocultural mechanisms through which emotions influence group identity (cohesion, resistance, reconstruction).	38	Studies exploring how emotions foster identity formation or collective action.

Table 4 summarizes the studies corresponding to Specific Objective 1, presenting the dominant emotions identified: nostalgia, guilt, shame, pride, hope, affection, resentment, and compassion. These emotions operate as mechanisms of cohesion, moral regulation, and symbolic agency, enabling a sense of belonging within contexts of mobility and inequality. Through them, individuals find ways to reinscribe their histories within a world that constantly redefines the boundaries of “us.”

**Table 4:** Most studied collective emotions.

**Primary emotion**	**Authors**	**Populations**	**Emotional findings**
Nostalgia	(Dounia, 2020; Kanouté and Lafortune, 2020; Kovats Sánchez, 2020; Laus, 2020; Marino, 2018; Pistrick, 2017; Smeekes and Jetten, 2019; Tuñón Pablos and Martínez Olvera, 2019)	Latin American, European, and African migrants in transnational mobility contexts	Nostalgia emerges as the dominant emotion of loss and belonging; it connects memory, identity, and culture through symbolic practices (food, music, rituals) that transform absence into resilience and cohesion.
Guilt	(Vermot, 2015)	Transnational Argentine families	A moral and gendered emotion that regulates family ties across distance, expressing tension between autonomy and care.
Shame/Stigma	(Dounia, 2020; Laus, 2020)	Racialized and indigenous migrants	Shame arises as a response to stigma and discrimination; its transformation into pride drives resistance and cultural self-affirmation.
Ethnic/Identity Pride	(Branch, 2021; Yazdiha, 2021)	Multicultural students and Muslim migrants	Reinforces intragroup cohesion, sense of belonging, and cultural affirmation in contexts of exclusion and Islamophobia.
Hope/Resilience	(Martinez-Damia et al., 2023; Nikielska-Sekuła, 2023)	Young European and Latin American migrants	Restorative emotions that promote cultural continuity, intergenerational connection, and community resilience.
Affection/Belonging	(Sanabria et al., 2024; Tsouroufli, 2023)	Students and professionals in multicultural settings	Affection fosters empathy and intercultural integration, strengthening emotional bonds among diverse groups.
Indignation/Resentment	(Castelo Branco and Gouvêa, 2025; Sánchez-Sibony, 2024)	Activism and social movements in Latin America	Resentment and indignation become moral energy that fuels collective action and resistance against injustice.
Compassion/Solidarity	(Elias et al., 2022)	Collective experiences of intercultural collaboration	Compassion emerges as a relational emotion that sustains solidarity and mutual support among diverse communities.

From Smeekes and Jetten (2019) only the second study was included, corresponding to first-generation immigrants in the Netherlands, since the first analyzed emotions among the majority group (native Dutch), which falls outside the population focus of this review.

Table 5, corresponding to Specific Objective 2, presents the institutional and political contexts examined. These findings reveal that emotions are not mere accompaniments to social life but structuring forces mediating between subjective experience and the organization of power. The analyzed contexts, educational, migratory, linguistic and political, constitute a field of emotional convergence where experiences of inequality, mobility and exclusion are transformed into bonds of symbolic repair and recognition. Shame, silence and anger are reconfigured into pride, trust and hope, expressing an emotional transition toward the moral reconstruction of the social bond. In this process, emotions emerge as devices of symbolic justice, capable of articulating memory, recognition and social transformation within communities that seek not merely to survive but to feel legitimately part of the world they inhabit.

**Table 5:** Social and institutional contexts.

**Context**	**Subcontext**	**Authors**	**Contextual findings**
Education	Intercultural and post-secondary university	(Aggarwal and Çiftçi, 2021; Branch, 2021; Elias et al., 2022; Gao, 2025; Gu et al., 2019; Gummadam et al., 2016; Kanouté and Lafortune, 2020; Kovats Sánchez, 2020; Sanabria et al., 2024; Tsang, 2022; Tsouroufli, 2023; Teyra et al., 2024; Yu et al., 2024)	Multicultural and intercultural university environments transform shame and silence into identity pride and academic agency, enabling classrooms and academic communities to function as spaces of symbolic repair, recognition, and dialog across knowledge systems.
Education	Intercultural school/classroom	(Almeida et al., 2023; An et al., 2022; Muller, 2016; Prehn et al., 2025; Uy, 2018)	Multicultural and multilingual classrooms foster emotional reflexivity and critical awareness of otherness, promoting empathy, group cohesion, and a shared sense of cultural belonging among students.
Migration/Diaspora	Transnational and digital community	(Cohen et al., 2025; Dounia, 2020; Mahmut, 2021; Tuñón Pablos and Martínez Olvera, 2019)	Migration activates processes of nostalgia and cultural grief; community and digital spaces enable the reconstruction of belonging through rituals, affective memory, and shared cultural practices.
Language/Linguistic Policy	Revitalization and bilingual programs	(Jo et al., 2023; Kovats Sánchez, 2020)	The revitalization and teaching of Indigenous or minority languages transform inherited shame into collective pride, re-signifying language as an emblem of cultural resistance, ethnic cohesion, and emotional wellbeing.
Activism/Social Movements	Feminist, anti-racist, and migrant collectives	(Asikainen and McAreavey, 2025; Boric Bargetto et al., 2021; Lund, 2024; Saco et al., 2022; Yazdiha, 2021)	Activism converts moral anger and resentment into solidarity, pride, and collective hope, shaping shared political identities that revalue diversity, denounce exclusion, and generate community agency.
Public Policy/Services	Integration, health, and recognition programs	(Feng and Zhu, 2022; Filomeno, 2019; Heid et al., 2022; Martinez-Damia et al., 2023; Nikielska-Sekuła, 2023; Zermeño and Laster Pirtle, 2021)	Inclusive institutional policies and support services foster hope, interethnic trust, and collective wellbeing, whereas the lack of recognition or participation reinforces feelings of exclusion, disaffection, and disconnection.

Table 6 introduces the sociocultural mechanisms identified across the reviewed studies, which explain how collective emotions influence processes of cohesion, resistance, and reconstruction of group identity. Each mechanism is associated with sub-processes of collective action and with representative authors providing recent empirical evidence. This systematization highlights the diversity of ways in which emotions operate across community, political, and symbolic levels, and how they articulate with the dynamics of belonging and cultural integration in both local and transnational contexts.

**Table 6:** Sociocultural mechanisms identified.

**Sociocultural mechanism**	**Subprocess or form of collective action**	**Authors**	**Summary of findings**
Emotional cohesion and belonging	Affective solidarity and intercultural empathy	(Branch, 2021; Gu et al., 2019; Sanabria et al., 2024; Tsouroufli, 2023)	Positive emotions such as affection, empathy, and shared pride strengthen group cohesion and intercultural integration; they foster support networks and a sense of belonging in academic and community spaces.
Symbolic resistance	Reappropriation of stigma and identity pride	(Castelo Branco and Gouvêa, 2025; Kovats Sánchez, 2020; Laus, 2020; Mahmut, 2021; Saco et al., 2022; Yazdiha, 2021)	Shame, resentment, and moral anger are transformed into pride and collective action, enabling communities to resist hegemonic narratives and construct political identities in response to discrimination.
Cultural reconstruction	Linguistic revitalization and re-signification of memory	(Dounia, 2020; Jo et al., 2023; Kovats Sánchez, 2020; Marino, 2018; Tuñón Pablos and Martínez Olvera, 2019)	Emotions such as nostalgia and hope drive cultural reconstruction and the intergenerational transmission of collective memory; rituals and language operate as axes of symbolic continuity.
Transnational community cohesion	Digital networks and rituals of belonging	(Boric Bargetto et al., 2021; Cohen et al., 2025; Kanouté and Lafortune, 2020)	Migrant communities use shared cultural practices (music, art, commemorations) to maintain transnational emotional ties, reinforcing collective identity beyond physical borders.
Political and moral resistance	Affective mobilization against injustice	(Asikainen and McAreavey, 2025; Castelo Branco and Gouvêa, 2025; Lund, 2024; Sánchez-Sibony, 2024)	Indignation and moral resentment generate collective action and structural resistance, transforming identity-related suffering into moral energy for social change.
Interethnic and inclusive reconstruction	Restorative emotions in public policies	(Feng and Zhu, 2022; Filomeno, 2019; Martinez-Damia et al., 2023; Heid et al., 2022; Nikielska-Sekuła, 2023; Zermeño and Laster Pirtle, 2021)	Recognition-based policies and community programs grounded in hope and interethnic trust act as emotional mechanisms for social reconstruction and reconciliation.
Moral cohesion and collective memory	Shared narratives of pain and hope	(Marino, 2018; Smeekes and Jetten, 2019; Vermot, 2015)	The evocation of collective experiences of loss and mourning generates moral cohesion, reinforcing historical identity, and the continuity of the group through affective memory.

## 4 Discussion

The systematic review demonstrates that collective emotions are not merely secondary affective responses but social and cultural practices that structure belonging and group identity. Among Indigenous, migrant, and minority populations, emotions such as nostalgia, pride, hope, and resentment operate as organizing forces of social bonds. This finding reinforces that cultural identity is sustained not only through discourse or institutions but also through shared affective climates that legitimize inclusion and exclusion (Ahmed, 2014; Barbalet, 2002; Martinez-Damia et al., 2023; Nikielska-Sekuła, 2023; Tuñón Pablos and Martínez Olvera, 2019).

### 4.1 Collective emotions as social structures of feeling

Across the narratives analyzed, emotions do not passively accompany identity processes; they shape, orient, and give meaning to how individuals understand their place within the group. They constitute the affective framework that sustains belonging and identity affirmation within migrant and Indigenous communities. Far from individual reactions, they function as shared patterns that structure social life. Among these, nostalgia occupies a central place. Its recurrent presence in the reviewed studies (Dounia, 2020; Kanouté and Lafortune, 2020; Kovats Sánchez, 2020; Laus, 2020; Marino, 2018; Pistrick, 2017; Smeekes and Jetten, 2019; Tuñón Pablos and Martínez Olvera, 2019) underscores its structuring role in transnational mobility processes. Beyond melancholy for the past, nostalgia functions as a symbolic reconstruction of home and affective continuity (Abakoumkin et al., 2020; Contini and Carrera, 2022).

This emotion transforms loss into connection and redefines distance as belonging, while shame may be rewritten as pride (Priwati and Sanitioso, 2024). For example, Marino (2018) shows how the Italian diaspora in London reconfigures everyday culinary narratives into sites of collective memory and symbolic continuity, whereas Tuñón Pablos and Martínez Olvera (2019) illustrate how Mexican migrants in New York transform nostalgic remembrance into an affective infrastructure of transnational cohesion. Memory materializes through sensory practices such as food, music, and everyday rituals that reactivate community and strengthen diasporic identities, turning remembrance into a form of cultural resistance (Smeekes and Jetten, 2019; Tuñón Pablos and Martínez Olvera, 2019). Guilt, though less frequent, illuminates the moral tensions between autonomy and care. In transnational families, it regulates affective ties at a distance, expressing the ethical conflict between the need to leave and the obligation to sustain family bonds (Vermot, 2015). More than an individual feeling, guilt acts as a symbolic mechanism of moral and emotional regulation. In Latin American contexts, family and community networks transform guilt into a relational resource that maintains connections with those who remain in the country of origin (Boric Bargetto et al., 2021; Gómez-Estern and de la Mata Benítez, 2013). From a sociological perspective, it manifests as an affective practice that renews belonging and reconfigures moral obligations of transnational care (Jardim and Marques da Silva, 2021; Liu et al., 2022).

Shame, a boundary emotion, marks both the wound of stigma and the starting point for its re-signification. Among racialized migrants and Indigenous groups, it emerges as a reaction to rejection and discrimination (Dounia, 2020; Laus, 2020). Within communities, however, shame transforms into pride and cultural affirmation, initiating a process of emotional politicization. Feelings once linked to subordination are rewritten into expressions of resistance and dignity (Ahmed, 2014; Illouz, 2019). As Gómez-Estern and de la Mata Benítez (2013) argue, migratory narratives rework displacement into processes of identity re-signification, transforming experiences of rupture into collective self-affirmation. This dynamic has been documented in diverse empirical contexts, including Latin American South–North migration to the United States and Southern European Diasporas in the United Kingdom, where narratives of mobility are rearticulated as moral and cultural continuity. This transition from shame to pride exemplifies how emotions not only reflect social positions but also produce symbolic shifts and new forms of recognition (Branch, 2021; Yazdiha, 2021). Ethnic and identity pride functions as a political emotion that articulates belonging and dignity. In contexts of exclusion, Islamophobia, or symbolic inequality, it operates as both an emotional response and a source of collective agency for racialized and minority communities confronting exclusion and symbolic inequality (Branch, 2021; Yazdiha, 2021). Within multicultural educational environments, pride becomes a means of resistance against hegemonic discourses of assimilation, enabling minority groups to define their identities from affirmation rather than deficit (Elias et al., 2022; Tsouroufli, 2023). It also strengthens collective self-esteem and interethnic recognition (Aggarwal and Çiftçi, 2021; Sanabria et al., 2024), demonstrating that emotional wellbeing depends on institutional recognition as much as on the reinforcement of collective pride.

Hope and resilience emerge as restorative emotions that enable the imagination of shared futures. Among young European and Latin American migrants, hope sustains cultural reconstruction in the face of displacement, acting as an affective horizon that provides continuity to identity (Feng and Zhu, 2022; Heid et al., 2022). These emotions imply an ethical disposition toward the future and the ability to rebuild social bonds amid conditions of exclusion and structural inequality (Filomeno, 2019; Zermeño and Laster Pirtle, 2021). Resilience integrates pain and resistance, transforming suffering into moral and collective agency (Martinez-Damia et al., 2023; Nikielska-Sekuła, 2023). Together, they express the capacity to project identity into the future as an act of resistance and cultural reaffirmation.

Affection and belonging constitute the emotional fabric that sustains intercultural relations. Recent studies conceptualize affection as a relational practice that fosters empathy, recognition, and moral cohesion (Elias et al., 2022; Sanabria et al., 2024; Tsouroufli, 2023). It serves as a symbolic bridge that dissolves hierarchies and generates inclusive spaces where difference is experienced as possibility (Gu et al., 2019; Tsang, 2022). This evidence reinforces that belonging is not achieved solely through structural integration but through affective ties that legitimize coexistence and emotional reciprocity.

Resentment and indignation act as moral engines of collective action (Asikainen and McAreavey, 2025; Castelo Branco and Gouvêa, 2025; Sánchez-Sibony, 2024). Both condense the discomfort provoked by injustice and transform it into political energy. Resentment can be understood as a form of inverted recognition, a reaffirmation of value in the face of systemic delegitimization (Lund, 2024). In feminist, anti-racist, and decolonial movements, these emotions express the memory of harm and articulate collective demands for justice, becoming a political language that redefines the boundaries of legitimacy (Castelo Branco and Gouvêa, 2025; Sánchez-Sibony, 2024).

Finally, compassion completes the emotional panorama as a relational feeling that sustains solidarity and mutual support (Elias et al., 2022; Sanabria et al., 2024). In intercultural contexts, compassion allows individuals to recognize the vulnerability of others as a reflection of their own humanity (Tsouroufli, 2023). This reciprocal recognition fosters cohesion, symbolic repair, and collective wellbeing. Rather than replacing justice, compassion precedes it, opening possibilities for structural change grounded in moral empathy and emotional reciprocity.

### 4.2 Institutional and political contexts of emotion

The reviewed studies reveal that collective emotions do not emerge in isolation but are embedded within institutional structures that give them meaning and direct their social function. Schools, universities, transnational communities, social movements, and public policies operate as arenas in which emotions are produced, legitimized, and transformed. Within these spaces, emotion ceases to be an individual reaction and becomes a social practice that organizes dynamics of recognition, agency, and symbolic justice.

In educational settings, emotions mediate processes of participation, belonging, and recognition. In multicultural universities and intercultural education environments, feelings such as shame or linguistic insecurity, historically associated with discrimination, are reconfigured into identity pride when institutions promote pedagogies grounded in empathy and dialogue (Aggarwal and Çiftçi, 2021; Sanabria et al., 2024; Tsouroufli, 2023). This transformation demonstrates that education not only transmits knowledge but also repairs symbolic and emotional inequalities. In multilingual contexts, classrooms operate as micro spheres of recognition where emotions make visible previously silenced trajectories and strengthen group cohesion (Kanouté and Lafortune, 2020; Prehn et al., 2025; Uy, 2018).

In contexts of migration and diaspora, collective emotionality manifests in transnational networks that sustain identity and memory. Studies by Marino (2018), Dounia (2020), Tuñón Pablos and Martínez Olvera (2019), and Cohen et al. (2025) show how nostalgia and cultural mourning are channeled through rituals, celebrations, and digital practices that preserve a sense of belonging. Digital platforms and community festivities reproduce affective ties and create emotional continuity among geographically dispersed individuals. In these contexts, emotion replaces territoriality as the source of cohesion, revealing that belonging depends more on shared affective intensity than on physical co-presence (Laus, 2020; Mahmut, 2021).

Language emerges as a transversal axis in the emotional configuration of communities. Processes of linguistic revitalization and bilingual education programs, as analyzed by Kovats Sánchez (2020) and Jo et al. (2023) re-signify language as an affective heritage and a symbol of cultural resistance. This reappropriation transforms historical stigma associated with Indigenous languages into collective pride, fostering ethnic cohesion and cultural continuity (Kanouté and Lafortune, 2020). The transition from fear and shame to linguistic pride underscores the affective relevance of language in contemporary processes of cultural decolonization (Kovats Sánchez, 2020).

Activism and social movements constitute spaces where emotion acquires an explicit political function. Research by Yazdiha (2021); Boric Bargetto et al. (2021); Asikainen and McAreavey (2025); Lund (2024); Saco et al. (2022) demonstrates that moral anger, resentment, and indignation can be transformed into solidarity, hope, and collective agency. In these spaces, emotion operates as a political pedagogy, teaching people to feel together, to recognize the pain of others, and to transform vulnerability into collective strength. Bodies in protest become visible expressions of the moral bond that unites mobilized communities. From this perspective, emotionality becomes both a language of social justice and a tool for redefining the boundaries of the (Castelo Branco and Gouvêa, 2025; Sánchez-Sibony, 2024).

Within the sphere of public policy, emotions act as sensitive indicators of inclusion or exclusion. Studies by Filomeno (2019); Zermeño and Laster Pirtle (2021); Heid et al. (2022); Feng and Zhu (2022); Nikielska-Sekuła (2023); Martinez-Damia et al. (2023) reveal that policies promoting recognition, participation, and equity generate restorative emotions such as hope, interethnic trust, and collective wellbeing. Conversely, exclusionary or bureaucratic policies intensify frustration, resentment, and social disaffection, undermining moral cohesion. These emotions function as barometers of the symbolic impact of public action, measuring how institutions are affectively perceived by communities and whether they repair or perpetuate the wounds of inequality. Collectively, the evidence suggests that collective emotional wellbeing constitutes a legitimate indicator of social justice and the symbolic efficacy of contemporary public policies (Heid et al., 2022; Martinez-Damia et al., 2023).

### 4.3 Sociocultural mechanisms: cohesion, resistance, and reconstruction

Collective emotions develop within institutional structures that give them meaning and shape their social function. Schools, universities, transnational communities, social movements, and public policies act as arenas where emotions are produced, legitimized, and transformed into social practices that regulate recognition, agency, and symbolic justice.

In educational contexts, emotions mediate processes of participation, belonging, and recognition. In multicultural universities and intercultural learning environments, feelings such as shame or linguistic insecurity, historically linked to discrimination, are transformed into identity pride when institutions foster pedagogies based on empathy and dialogue (Aggarwal and Çiftçi, 2021; Sanabria et al., 2024; Tsouroufli, 2023). This reconfiguration demonstrates that education not only transmits knowledge but also repairs symbolic and emotional inequalities. In multilingual contexts, classrooms operate as micro-spheres of recognition where emotions make visible previously silenced trajectories and reinforce group cohesion (Kanouté and Lafortune, 2020; Uy, 2018; Prehn et al., 2025).

In contexts of migration and diaspora, transnational networks function as emotional spaces of identity continuity. Recent research (Marino, 2018) shows how nostalgia and cultural mourning are channeled through rituals, celebrations, and digital platforms that recreate belonging. In such environments, emotion replaces territoriality as the source of cohesion, revealing that belonging depends more on the intensity of shared affect than on physical co-presence (Laus, 2020; Mahmut, 2021).

Language constitutes a transversal axis in the emotional experience of communities. Processes of linguistic revitalization and bilingual education re-signify language as affective heritage and a symbol of cultural resistance (Jo et al., 2023; Kovats Sánchez, 2020), reflecting a shift from fear or shame to linguistic pride in contemporary processes of cultural decolonization.

At a mechanistic level, these dynamics illustrate how moral emotions function as catalysts of collective mobilization. Anger, resentment, and indignation operate as affective triggers that convert experiences of injustice into shared moral claims, transforming emotional energy into organized political agency and redefining the boundaries of legitimacy (Asikainen and McAreavey, 2025; Castelo Branco and Gouvêa, 2025; Sánchez-Sibony, 2024).

Finally, public policies reveal how emotions act as sensitive indicators of inclusion or exclusion. Filomeno (2019) shows that policies grounded in recognition and participation generate restorative emotions such as hope and interethnic trust, a pattern also observed in subsequent studies (Zermeño and Laster Pirtle, 2021; Heid et al., 2022; Feng and Zhu, 2022; Nikielska-Sekuła, 2023; Martinez-Damia et al., 2023). Conversely, exclusionary policies reinforce frustration and social disengagement. At the level of social mechanisms, this dynamic reveals how emotional responses to institutional recognition or exclusion become internalized as moral evaluations of legitimacy, shaping collective perceptions of justice, and belonging.

### 4.4 Strengths, limitations, and sociological implications

This review is distinguished by its strength in integrating empirical and theoretical evidence from a sociological perspective that transcends traditional psychological frameworks in the study of collective emotions. The use of PRISMA guidelines, the inclusion of high-impact databases (Scopus, Web of Science, and ScienceDirect), and the application of rigorous selection criteria ensure methodological validity and transparency. However, the heterogeneity of approaches among the reviewed studies, with a predominance of qualitative methodologies and limited standardization of sociological indicators of affect, restricts the possibility of quantitative comparisons or generalizations. Moreover, the underrepresentation of research from non-Western regions limits the epistemological diversity of the field. Despite these constraints, the findings provide significant implications for contemporary sociology by demonstrating that collective emotions are not merely individual phenomena but social practices that structure belonging, agency, and symbolic justice. In this regard, there is a need to advance toward a sociology of collective emotions capable of articulating emotion, identity, and power within contexts of globalization, inequality, and cultural reconstruction.

### 4.5 Projections and future research directions

The findings of this review open new avenues for the sociology of emotions by demonstrating that the study of collective emotions provides critical insights into processes of cohesion, resistance, and cultural reconstruction within contexts of mobility, inequality, and social transformation. Future research could explore how emotions shape moral infrastructures that sustain social cohesion, particularly within communities affected by displacement, coloniality, or structural inequality. It is also necessary to advance methodological frameworks that integrate emotional analysis with intersectional and epistemic justice approaches, allowing for a deeper understanding of how collective emotions operate as political and cultural forces of resistance.

In particular, the integration of ethnographic, discursive, and neuro-affective perspectives could illuminate the mechanisms through which emotional memory and belonging are reconfigured in contexts of migration and digital globalization. Finally, comparative studies among Indigenous, rural, and urban communities would offer a broader understanding of the ways in which collective emotions contribute to the reconstruction of the social fabric and to the creation of shared futures grounded in empathy, dignity, and moral reciprocity.

## 5 Conclusions

The analysis of empirical and theoretical evidence shows that collective emotions form the invisible fabric that sustains the cultural identity, memory, and resilience of Indigenous and minority groups. Through them, communities of meaning emerge in which belonging and dignity are reconstructed in the face of exclusion. Far from being individual reactions, emotions function as social structures of feeling that mediate between memory, culture, and power, articulating cohesion, resistance, and reconstruction. Emotions such as nostalgia, pride, hope, and compassion operate as mechanisms of symbolic continuity and moral repair, while shame and resentment—originally linked to stigma and exclusion—are re-signified as emotions of identity affirmation and moral energy for collective action. In educational, community, and political settings, these emotions act as forces of integration and symbolic justice, enabling communities to reinscribe their history and dignity within a constantly changing world.

This systematic review contributes to advancing and systematizing an emerging field within the sociology of emotions while also providing a replicable methodological framework for the comparative study of collective emotions in intercultural contexts.

## Data Availability

Publicly available datasets were analyzed in this study. This data can be found here: 10.5281/zenodo.17873399.
